# Supplementary material for: Advances in Pharmacological Properties, Molecular Mechanisms, and Bioavailability Strategies of Chlorogenic Acid in Cardiovascular Diseases Therapy
Source: Pharmaceuticals (Basel). 2025 Sep 11;18(9):1357. doi: 10.3390/ph18091357 (PMC12472252; doi:10.3390/ph18091357)
Supplement: Supplementary file 1 [file pharmaceuticals-18-01357-s001.zip › pharmaceuticals-3795302-supplementary.pdf]

Supplemental Material

Advances in Pharmacological Properties, Molecular Mechanisms, and Bioavailability Strategies of Chlorogenic Acid in Cardiovascular Diseases Therapy

Kai Huang [1,†], Duosu Zhang [1,†], Ruting Wang [1], Jiahao Duan [1], Long Hu [1], Fan Huang [1], Wei Liu [1], Jia Gu [1], Songlin Li [2], Chun Yang [3,*] and Ling Yang [1,*]

[1] Department of Cardiology, The Third Affiliated Hospital of Soochow University, Changzhou 213003, China
[2] Department of Cardiology, Changzhou Medical Center, Nanjing Medical University, Changzhou 213003, China
[3] Department of Anesthesiology and Perioperative Medicine, The First Affiliated Hospital of Nanjing Medical University, Nanjing 210029, China
[*] Correspondence: chunyang@njmu.edu.cn (C.Y.); yangling369@czfph.com (L.Y.)
[†] These authors contributed equally to this work.

.

Table S1. Summary of clinical trials related to CGA.

Author, year	Study Design	Participants, n	Sex	Age (mean)	Duration	CGA Dose	CGA Source	Significant outcomes
Olthof et al. 2001 [66]	Crossover	20	10M/10F	24 ± 8	4 weeks	2000 mg	Coffee	Increased postprandial plasma total homocysteine (+12%); Increased fasting plasma total homocysteine (+4%); Decreased fasting plasma folate concentration decreased (-8%)
Agudelo-Ochoa et al. 2016 [24]	Randomized, Single-blind, Placebo-controlled	Int1: 25 Int2: 24 Con: 25	38M/37F	38.5 ± 9.0	8 weeks	Int1: 420 mg/day Int2: 780 mg/day	Filtered Coffee	Increased plasma antioxidant capacity
Kajikawa et al. 2019 [25]	Randomized, Single-blind, Placebo-controlled, Crossover	Int: 9 Con: 9	12M/6F	56.0 ± 15.0	Acute effects measured over 2 hours	412 mg	Roasted coffee with absorption purification process	Increased Flow-Mediated Dilation; Decreased 8-isoprostane levels
Suzuki et al. 2019 [32]	Double-blind, Placebo-controlled, Pilot study	Int: 8 Con: 8	16M/0F	44.6 ± 5.3	2 weeks	300 mg/day	Green coffee beans	Improved arterial stiffness; Increased Flow-mediated dilation; Decreased Sympathetic Nervous Activity
Lara-Guzmán et al. 2020 [63]	Randomized, Single-blind, Placebo-controlled	Int1: 24 Int2: 25 Con: 25	37M/37F	38.0 ± 9.0	8 weeks	Int1: 787 mg/day; Int2: 407 mg/day	Coffee beverage (Colombian	Reduced urinary oxylipins (IsoPs, PGs, and their metabolites)

							Arabica varieties)	
Lara-Guzmán et al. 2021 [29]	Randomized, Placebo-controlled	Int1: 24<br>Int2: 25<br>Con: 25	37M/37F	Int1: 38.04 ± 8.74<br>Int2: 38.00 ± 8.87<br>Con: 38.04 ± 9.99	8 weeks	Int1: 787 mg/day;<br>Int2: 407 mg/day	Colombian Arabica coffee (light and dark roast)	Increased cholesteryl esters (+9%); Decreased oxysterols (−71%) and free fatty acids (−29%)
